# Pneumocystis pneumonia in patients with rheumatic diseases receiving prolonged, non-high-dose steroids—clinical implication of primary prophylaxis using trimethoprim–sulfamethoxazole

**DOI:** 10.1186/s13075-019-1996-6

**Published:** 2019-09-14

**Authors:** Jun Won Park, Jeffrey R. Curtis, Min Jung Kim, Hajeong Lee, Yeong Wook Song, Eun Bong Lee

**Affiliations:** 1 Division of Rheumatology, Department of Internal Medicine, Seoul National University College of Medicine, 101 Daehak-ro, Jongno-gu, Seoul, 03080 Republic of Korea; 2 Division of Clinical Immunology & Rheumatology, University of Alabama at Birmingham, Birmingham, AL USA; 3 Division of Nephrology, Department of Internal Medicine, Seoul National University College of Medicine, Seoul, Republic of Korea

**Keywords:** Pneumocystis pneumonia, Glucocorticoids, Trimethoprim–sulfamethoxazole, Prophylaxis

## Abstract

**Objectives:**

To investigate the incidence of pneumocystis pneumonia (PCP) and its risk factors in patients with rheumatic disease receiving non-high-dose steroid treatment, along with the risks and benefits of PCP prophylaxis.

**Methods:**

This study included 28,292 treatment episodes with prolonged (≥ 4 weeks), non-high-dose steroids (low dose [< 15 mg/day, *n* = 27,227] and medium dose [≥ 15 to < 30 mg/day, *n* = 1065], based on prednisone) over a 14-year period. Risk factors for PCP and prophylactic effect of trimethoprim–sulfamethoxazole (TMP-SMX) were investigated if the 1-year incidence rate (IR) of PCP in each dose group was > 0.1/100 person-years. Cox regression with LASSO was used for analysis.

**Results:**

One-year PCP IR in the low-dose group was 0.01 (95% CI 0.001–0.03)/100 person-years, and only the medium-dose group showed eligible PCP IR for further analysis. In the medium-dose group, prophylactic TMP-SMX was administered in 45 treatment episodes while other episodes involved no prophylaxis (prophylaxis group vs. control group). In 1018.0 person-years, 5 PCP cases occurred exclusively in the control group, yielding an IR of 0.5 (0.2–1.2)/100 person-years. Concomitant steroid-pulse treatment and baseline lymphopenia were the most significant risk factors for PCP. Treatment episodes with at least one of these factors (*n* = 173, high-risk subgroup) showed higher 1-year PCP IR (3.4 (1.1–8.0)/100 person-years), while no PCP occurred in other treatment episodes. TMP-SMX numerically reduced the risk (adjusted HR = 0.2 (0.001–2.3)) in the high-risk subgroup. The IR of adverse drug reactions (ADRs) related to TMP-SMX was 41.5 (22.3–71.6)/100 person-years, including one serious ADR. The number needed to treat with TMP-SMX to prevent one PCP in the high-risk subgroup (31 (17–226)) was lower than the number needed to harm by serious ADR (45 (15–∞)).

**Conclusion:**

Incidence of PCP in patients with rheumatic diseases receiving prolonged, medium-dose steroids depends on the presence of risk factors. Prophylactic TMP-SMX may have greater benefit than potential risk in the high-risk subgroup.

## Background

Pneumocystis pneumonia (PCP) is a potentially life-threatening infectious disease that mainly occurs in immunocompromised hosts [1]. PCP was first recognized as the most common opportunistic infection in patients infected with human immunodeficiency virus (HIV), but its incidence has fallen dramatically since the development of effective anti-retroviral treatment [2]. However, PCP is still an important cause of atypical pneumonia in patients without HIV who have immunosuppressed conditions [3, 4].

Incidence of PCP in patients with rheumatic diseases remains uncertain. Previous study reported that its prevalence in autoimmune diseases was 1~7% in systemic lupus erythematosus (SLE), 2~37.5% in inflammatory myositis, and 6~17% in Wegener’s granulomatosis [5]. The broad range of prevalence among studies suggests that incidence of PCP could be different according to immunosuppressant use. However, all of these studies consistently reported high PCP-related mortality, which spanned between 30 and 80%. Considering that PCP in patients without HIV usually follows a fulminant course and has higher mortality than PCP in HIV-infected patients, evidence on incidence and efficacy of primary prophylaxis of PCP is required for better treatment outcome of rheumatic diseases [6–9].

The most significant risk factor for PCP in patients without HIV is treatment with a moderate-to-high dose of glucocorticoid, which is the principal therapeutic agent for many rheumatic diseases [6, 10]. We previously demonstrated that prophylactic trimethoprim–sulfamethoxazole (TMP-SMX) significantly decreases the incidence of PCP in patients with rheumatic diseases who receive prolonged (≥ 4 weeks), high-dose (≥ 30 mg/day prednisone) steroid treatment, with an acceptable safety profile [11]. However, it is still uncertain whether prophylaxis is justified in patients who receive lower doses of glucocorticoid treatment. The incidence of PCP and its potential risk factors have not been adequately characterized in this group of patients, and reports have been limited to small case series and observational studies [12–15].

The main objective of this study was to identify the risk factors which primary PCP prophylaxis is justified despite relatively lower dose of steroid treatment. We determined the incidence of PCP in patients with rheumatic diseases receiving various doses of steroids for ≥ 4 weeks and investigated the clinical features that affected the incidence of PCP.

## Methods

### Patients and treatment episodes

The electronic medical database at Seoul National University Hospital was analyzed for data collection and capturing study population. The institute is one of the largest tertiary referral centers in South Korea and covers patients living in all geographic areas of the country. The database was reviewed for the period between January 2004 and December 2017 to capture the “treatment episode,” which was defined as a clinical situation where a patient received steroid treatment within a specific dose range for ≥ 4 consecutive weeks. First, we collected the entire prescription data of oral/IV steroids. Then, according to the initial 4-week dose of steroid and prednisone-equivalent dose conversions, ≥ 2.5 to < 15 mg/day was defined as low dose, ≥ 15 to < 30 mg/day as medium dose, and ≥ 30 mg/day as high dose. The definition was based on the degree of steroid receptor saturation and previous studies reporting PCP cases in patients with steroid treatment [6, 10, 12, 16, 17]. All captured episodes were then linked to the database of registered ICD-10 code of all patients in our institution. If a patient had more than one ICD-10 codes of rheumatic diseases, the patient’s medical records were reviewed to confirm the diagnostic code.

The starting date of each treatment episode was defined as the first day of particular dose of steroid treatment. The observation period following each treatment episode was 1 year, unless PCP or a censoring event (death or loss to follow-up) occurred. To enable precise determination of the effect of initial steroid treatment, multiple episodes in a single patient had to be spaced ≥ 1 year apart to be included in the study. Defining process of treatment episodes is summarized in Additional file 1: Figure S1. Patients with a history of PCP, HIV infection, current cancer, or a solid organ transplant, and those < 18 years old, were excluded. The ICD-10 codes for case identification are presented as Additional file 1.

Incidence of PCP in each dose group was determined, and the values were compared. If the incidence rate (IR) was < 0.1 per 100 person-years, the risk from PCP prophylaxis (previously calculated as the incidence of serious adverse drug reaction (ADR) related to TMP-SMX) [11] was considered to clearly outweigh any potential benefit. Therefore, the efficacy of primary PCP prophylaxis was only assessed for PCP IR ≥ 0.1 per 100 person-years. PCP IR in the high-dose group was used for only comparison with that in each non-high-dose group.

Treatment episodes in the eligible dose group were classified into two groups (control or prophylaxis group) according to whether a patient received primary PCP prophylaxis concomitant with initiation of glucocorticoid treatment. The baseline date was the first day of specific dose of glucocorticoid treatment (control group) or TMP-SMX (prophylaxis group). Each patient received the corresponding dose of steroid for ≥ 4 weeks from the baseline date.

The primary outcome was the 1-year incidence of PCP in each group. Secondary outcome was the incidence of ADRs related to the prophylaxis. All suspected ADRs were reviewed by two authors (JWP and MJK) and assigned a probability of causation based on their timing compared with established patterns of specific ADRs. Among them, only those with probable/likely or certain causality were finally selected [18].

The study was carried out in accordance with the Declaration of Helsinki and was approved by the institutional review board (IRB) of Seoul National University Hospital [IRB No 0907-062-287]. Consent was waived by the IRB because of the retrospective nature of the study.

### Detection of clinically relevant PCP

An algorithm was constructed to enable detection of symptomatic PCP occurring during the observation period in patients with rheumatic disease (Additional file 1: Figure S2). All data for confirmatory microbiological tests for PCP (polymerase chain reaction and direct fluorescent antibody staining of induced sputum or bronchoalveolar lavage fluid) conducted from January 2004 to December 2017 were obtained from medical records. Medical records of patients with positive test results were further evaluated to ascertain whether they had clinical features of PCP infection. All diagnoses of PCP were confirmed based on (1) positive PCR and/or immunofluorescence result of induced sputum or bronchoalveolar lavage fluid, (2) whether the patient showed appropriate clinical features of PCP (acute onset of dyspnea, cough, or fever, along with characteristic radiographic findings), and (3) exclusion of other pulmonary infections. Characteristic radiographic findings of PCP were bilateral interstitial infiltrates, predominant in the perihilar regions and the apices [19]. If a patient showed typical manifestations suggesting PCP but chest radiographs were inconclusive, high-resolution computerized tomography (HRCT) was evaluated to confirm the diagnosis. Positive microbiological tests without clinical manifestations consistent with respiratory infection were not considered to indicate PCP.

### PCP prophylaxis

TMP-SMX was the only agent used for PCP prophylaxis in this study. Because there was no consensus regarding PCP prophylaxis in patients with rheumatic disease receiving steroid treatment, initiation and duration of prophylaxis was mainly determined by physicians’ judgment. In the prophylaxis group, patients started TMP-SMX concurrently with steroid treatment unless they had contraindications for use of TMP-SMX at this time. TMP-SMX was given as one single-strength tablet (400/80 mg) per day, and the dose was adjusted according to the patient’s creatinine clearance (one tablet three times weekly when creatinine clearance 15~30 mL/min). Second-line prophylactic agents such as dapsone, atovaquone, and aerosolized pentamidine were not used for primary prophylaxis against PCP during the observation period.

### Statistical analysis

Continuous and dichotomous variables were analyzed using Student’s *t* test and the chi-square test, respectively. The Cox proportional-hazards regression model was used for comparison of the incidence of PCP between the groups and for estimation of effects of clinical factors on the 1-year PCP incidence. The hazard ratio (HR) was adjusted for baseline clinical factors that showed a relevant association (*p* < 0.1) with outcome. If an outcome variable showed a complete separation, Firth’s penalized maximum likelihood was used for reduction of statistical bias [20]. In addition, the final model was adjusted for intra-cluster correlation using grouped sandwich variance estimates, as some patients may have undergone multiple treatment episodes. To define the high-risk subgroup for PCP, a penalized regression method with the least absolute shrinkage and selection operator (LASSO) was used to select variables that predict the outcome most precisely. Briefly, among the nine variables (age, sex, disease duration, initial steroid dose, concomitant cyclophosphamide treatment, high cumulative steroid use, interstitial lung disease, lymphopenia, and concomitant steroid pulse) potentially associated with PCP, cross-validation was used to find the model with the minimal value for tuning parameter lambda. Because all clinical variables used for analysis had less than 1% of missing data, all statistical analyses were performed without imputation of them.

All statistical analyses were performed using R 3.3.1 software (package glmnet for Cox regression with LASSO), and a *p* value < 0.05 was considered statistically significant.

## Results

### Incidence of PCP with different steroid treatment doses

**Fig. 1:**
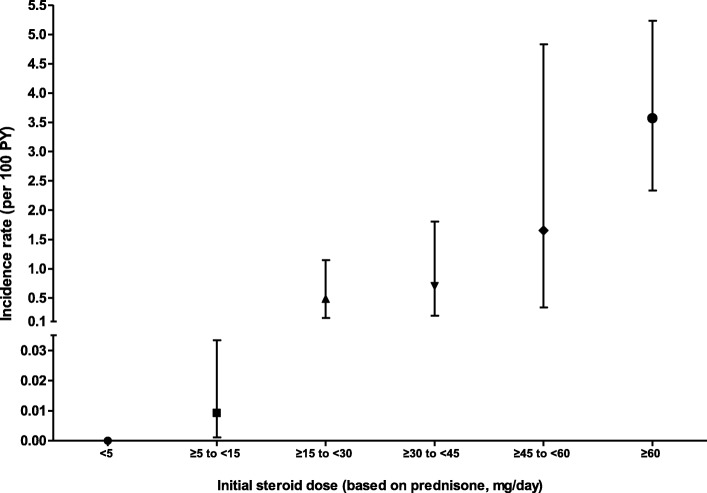
The 1-year incidence rates of pneumocystis pneumonia in treatment episodes with various ranges of steroid doses. Notably, the incidence was considerably higher for daily doses of steroids ≥ 15 mg of prednisone or equivalent. Error bar indicates the upper margin of the 95% confidence interval of the incidence rate

A total of 28,292 treatment episodes with non-high-dose steroid (27,227 low-dose group and 1065 medium-dose group) and 1665 episodes with high-dose steroid were initially analyzed. The 1-year IR of PCP increased progressively with increasing daily steroid dose (Fig. 1). In the treatment episodes with < 15 mg/day prednisone or equivalent, the IR was much lower than the pre-defined threshold value of 0.1 per 100 person-years. Therefore, the efficacy of TMP-SMX for primary PCP prophylaxis was only assessed in the medium-dose group.

### Patient characteristics of the medium-dose group

A total of 1065 treatment episodes in 732 rheumatic patients with prolonged, medium-dose steroid treatment fulfilled the criteria for analysis (Additional file 1: Figure S3). Baseline characteristics for this group are shown in Table 1. In this cohort, SLE was the most common disease (44.4%), followed by Behcet’s disease (19.7%). In the 89 (8.4%) treatment episodes, patient had interstitial lung disease (ILD) proven in chest computerized tomography. Concomitant steroid-pulse treatment at baseline was performed in 5.1% of the cohort. In the 83 (7.8%) treatment episodes, observation was censored because of follow-up loss (*n* = 66), death (*n* = 12), or PCP (*n* = 5).

**Table 1:** Baseline characteristics of the medium-dose treatment episodes

*n* = number of treatment episodes	Overall (*n* = 1065)	Control group (*n* = 1020)	Prophylaxis group (*n* = 45)	*p* ^a^
Age (years), mean (SD)	43.4 (15.0)	43.2 (14.9)	47.4 (17.7)	0.072
Male sex, *n* (%)	296 (27.8)	281 (27.5)	15 (33.3)	0.397
Disease duration (years), mean (SD)	5.1 (4.5)	5.2 (4.5)	3.9 (5.0)	0.057
Underlying disease				
Systemic lupus erythematosus, *n* (%)	473 (44.4)	458 (44.9)	15 (33.3)	0.126
Systemic sclerosis, *n* (%)	22 (2.1)	21 (2.1)	1 (2.2)	0.940
Polymyositis, *n* (%)	57 (5.4)	55 (5.4)	2 (4.4)	0.782
Dermatomyositis, *n* (%)	59 (5.5)	50 (4.9)	9 (20.0)	< 0.001
GPA, *n* (%)	6 (0.6)	0 (0.0)	6 (13.3)	< 0.001
MPA, *n* (%)	3 (0.3)	1 (0.1)	2 (4.4)	< 0.001
EGPA, *n* (%)	29 (2.7)	28 (2.7)	1 (2.2)	0.833
Polyarteritis nodosa, *n* (%)	8 (0.8)	7 (0.7)	1 (2.2)	0.243
Rheumatoid arthritis, *n* (%)	67 (6.3)	64 (6.3)	3 (6.7)	0.916
Adult-onset Still’s disease, *n* (%)	28 (2.6)	27 (2.6)	1 (2.2)	0.862
Behcet’s disease, *n* (%)	210 (19.7)	209 (20.5)	1 (2.2)	0.003
Ankylosing spondylitis, *n* (%)	16 (1.5)	16 (1.6)	0 (0.0)	0.397
Primary Sjogren’s syndrome, *n* (%)	14 (1.3)	13 (1.3)	1 (2.2)	0.585
Relapsing polychondritis, *n* (%)	12 (1.1)	11 (1.1)	1 (2.2)	0.477
Polymyalgia rheumatica, *n* (%)	21 (2.0)	21 (2.1)	0 (0.0)	0.331
Giant-cell arteritis, *n* (%)	2 (0.2)	2 (0.2)	0 (0.0)	0.766
Takayasu’s arteritis, *n* (%)	28 (2.6)	28 (2.7)	0 (0.0)	0.260
Others, *n* (%)^b^	10 (0.9)	9 (0.9)	1 (2.2)	0.362
Concomitant immunosuppressive treatment				
Steroid-pulse treatment, *n* (%)	54 (5.1)	42 (4.1)	12 (26.7)	< 0.001
Oral cyclophosphamide, *n* (%)	18 (1.7)	14 (1.4)	4 (8.9)	< 0.001
Cyclophosphamide pulse, *n* (%)	38 (3.6)	29 (2.8)	9 (20.0)	< 0.001
Azathioprine, *n* (%)	236 (22.2)	225 (22.1)	11 (24.4)	0.706
Mycophenolate mofetil, *n* (%)	184 (17.3)	181 (17.7)	3 (6.7)	0.054
Methotrexate, *n* (%)	169 (15.9)	167 (16.4)	2 (4.4)	0.032
TNFi, *n* (%)	20 (1.9)	18 (1.8)	2 (4.4)	0.195
Cumulative steroid dose, mean (SD)^c^	681.3 (1306.0)	657.1 (1267.5)	1229.8 (1928.5)	0.055
Interstitial lung disease, *n* (%)	89 (8.4)	73 (7.1)	17 (37.8)	< 0.001
Lymphopenia, *n* (%)^d^	131 (12.3)	123 (12.1)	8 (17.8)	0.253

The baseline date was defined as the day on which PCP prophylaxis (prophylaxis group) or medium-dose steroid (control group) was started

*GPA* granulomatosis with polyangiitis, *MPA* microscopic polyangiitis, *EGPA* eosinophilic granulomatosis with polyangiitis, *SD* standard deviation, *TNFi* tumor necrosis factor inhibitor

^a^*p* values for comparison of parameters between the control group and the prophylaxis group

^b^Including mixed connective tissue disease, IgG4-related disease and hypersensitivity vasculitis

^c^Cumulative steroid (prednisone) dose during the previous 6 months

^d^Defined as < 800 lymphocytes per microliter

TMP-SMX prophylaxis was performed in 45 (4.2%) treatment episodes, with a mean (SD) duration of 290 (275) days. Renal dose adjustment of TMP-SMX was performed in 7 episodes. Prophylaxis was initiated at the same time as the steroid treatment in 43 episodes, and in 2 episodes, the intended TMP-SMX initiation was delayed for 2 days for a missed prescription and for 19 days for leukopenia. Baseline characteristics of the control and prophylaxis groups differed significantly for several variables (Table 1). Patients in the prophylaxis group were more likely to have dermatomyositis, granulomatosis with polyangiitis, or microscopic polyangiitis and less frequently had Behcet’s disease. Concomitant steroid pulse (250~1000 mg/day methylprednisolone for 1~3 days) and cyclophosphamide were used more frequently in the prophylaxis group. However, the mean cumulative steroid dose administered during the entire observation period was comparable between the two groups (prednisone equivalent, 4385.0 ± 2037.1 vs. 4405.7 ± 2337.8 mg, *p* = 0.947). In contrast, mycophenolate mofetil (MMF) and methotrexate (MTX) were more frequently used in the control group. Other immunosuppressive agents such as azathioprine, cyclosporine, and tumor necrosis factor inhibitor (TNFi) were comparably used between the two groups. The proportion of treatment episode with baseline ILD was also higher in the prophylaxis group (7.1% vs. 37.8%).

### Incidence of PCP and risk factors in the medium-dose group

During a total of 1018.0 person-years, five PCP cases occurred, giving an IR of 0.5 (95% CI, 0.2–1.2) per 100 person-years. Clinical features of each PCP case at baseline and at the time of PCP diagnosis are presented in Additional file 1: Table S1. All cases occurred in the control group within the first 6 months of the observation period, and two individuals died as a result of PCP.

The results of univariable Cox regression analysis showed that concomitant steroid pulse at baseline was the most significant risk factor for PCP (HR = 75.0; 95% CI, 8.4–671.4). Baseline lymphopenia (< 800 lymphocytes/μL) and concomitant cyclophosphamide treatment (oral or intravenous) were also risk factors for PCP. High cumulative steroid (≥ 900 mg of prednisone or equivalent in the 6 months prior to the baseline date) was associated with increased risk for PCP, although it was not statistically significant (*p* = 0.084).

**Fig. 2:**
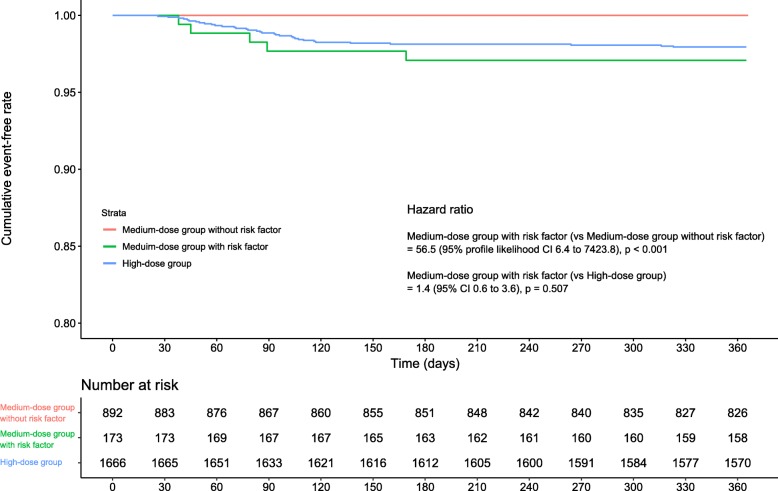
Kaplan–Meier curves indicating PCP-free survival in groups with medium-dose and high-dose groups. The medium-dose group is further stratified into a high-risk subgroup, with the presence of at least one risk factor (baseline lymphopenia and/or concomitant steroid pulse), and a non-high-risk subgroup, with no risk factors

In the multivariable analysis where these clinical factors were included as covariates, only concomitant steroid-pulse treatment and baseline lymphopenia were significantly associated with the incidence of PCP (Table 2). Model selection using LASSO regression also demonstrated that the model including steroid pulse and lymphopenia was the best model for precise prediction of PCP occurrence in the medium-dose population (Additional file 1: Figure S4). On the basis of these results, 173 treatment episodes where the patient had lymphopenia and/or had received concomitant steroid-pulse treatment were classified as a high-risk subgroup. All treatment episodes in which PCP occurred were in this subgroup. The 1-year IR for PCP in the high-risk subgroup was 3.4 (95% CI, 1.1–8.0) per 100 person-years, which was significantly higher than that in the remaining treatment episodes (HR = 56.5; 95% profile likelihood CI, 6.4–7423.8). It was also comparable to that in the high-dose group (Fig. 2).

**Table 2:** Clinical factors affecting 1-year PCP incidence

	Univariable analysis		Multivariable analysis^a^	
	HR (95% CI)	*p* value	Adjusted HR (95% CI)	*p* value
Old age (≥ 70 years old)	4.4 (0.5–39.6)	0.183	b	
Male sex	1.5 (0.2–13.7)	0.702	b	
Disease duration (years)	0.9 (0.7–1.2)	0.482	b	
Initial steroid dose, mg (based on prednisone)	1.1 (0.9–1.4)	0.377	b	
Concomitant steroid-pulse treatment	75.0 (8.4–671.4)	< 0.001	68.4 (5.3–876.0)	0.001
Concomitant cyclophosphamide treatment	12.2 (2.0–72.8)	0.006	1.5 (0.2–10.5)	0.707
Concomitant azathioprine	0.9 (0.1–7.8)	0.904	b	
Concomitant MMF	1.2 (0.1–10.6)	0.881	b	
Concomitant MTX	3.5 (0.6–21.1)	0.167	b	
Concomitant TNFi	4.6 (0.03–40.8)	0.395	b	
High cumulative steroid dose^c^ (≥ 900 mg)	4.9 (0.8–29.0)	0.084	1.7 (0.1–4.8)	0.610
Interstitial lung disease	2.8 (0.3–24.7)	0.363		
Baseline lymphopenia^d^	10.7 (1.8–63.8)	0.010	6.3 (1.01–39.1)	0.049

*CI* confidence interval, *HR* hazard ratio, *MMF* mycophenolate mofetil, *MTX* methotrexate, *PCP* pneumocystis pneumonia, *TNFi* tumor necrosis factor inhibitor

^a^Model included clinical factors that showed significant association (*p* < 0.1) in the univariable analysis, and was adjusted for clustering

^b^Not included in the multivariable model as a covariate

^c^Cumulative steroid (prednisone) dose during the previous 6 months

^d^Defined as < 800 lymphocytes per microliter

### Efficacy of TMP-SMX prophylaxis

The results of the Cox proportional hazard model in the medium-dose group indicated that TMP-SMX prophylaxis did not significantly decrease the 1-year PCP IR (adjusted HR = 0.5; 95% profile likelihood CI, 0.004–5.3). However, in the high-risk subgroup, TMP-SMX numerically reduced the 1-year PCP IR, although this effect was not statistically significant (adjusted HR = 0.18; 95% profile likelihood CI, 0.001–2.3) (Table 3). To investigate other populations in which PCP prophylaxis might be beneficial, the effect of TMP-SMX on 1-year PCP IR was estimated in specific subgroups. However, none of the subgroup analyses showed a significant decrease in PCP IR in association with prophylaxis (Additional file 1: Table S2).

**Table 3:** Effect of TMP-SMX prophylaxis on 1-year PCP incidence stratified according to the presence of risk factors

	Whole population (*n* = 1065)		High-risk subgroup^a^ (*n* = 173)	
	Hazard ratio (95% profile likelihood CI)		Hazard ratio (95% profile likelihood CI)	
	Univariable analysis	Multivariable analysis^b^	Univariable analysis	Multivariable analysis^c^
TMP-SMX prophylaxis	2.03 (0.02–17.95)	0.51 (0.004–5.30)	0.76 (0.006–6.74)	0.18 (0.001–2.31)
*p* value for hazard ratio	0.661	0.627	0.850	0.216

*CI* confidence interval, *PCP* pneumocystis pneumonia, *TMP-SMX* trimethoprim–sulfamethoxazole

^a^Defined as treatment episodes with baseline lymphopenia and/or concomitant steroid-pulse treatment

^b^Included concomitant steroid pulse and cyclophosphamide, higher previously used steroid (≥ 800 mg of prednisone or equivalent during previous 6 months) and lymphopenia as covariates, and was also adjusted for clustering

^c^Included old age (≥ 70 years) and concomitant steroid-pulse treatment as covariates and was also adjusted for clustering

### Safety and risk–benefit assessment of prophylactic TMP-SMX

During the 35.8 person-years of TMP-SMX prophylaxis (in 45 treatment episodes), 12 ADRs occurred in 10 treatment episodes, with an IR of 33.5 (95% CI, 17.3–58.5) per 100 person-years (Table 4). Abnormal results in liver function tests were the most common ADR, with an IR of 8.4 (95% CI, 1.7–24.5 per 100 person-years), followed by skin rash and thrombocytopenia, both with an IR of 5.6 (95% CI, 0.7–20.2) per 100 person-years. Most ADRs showed mild-to-moderate severity (11/12, 91.7%), but TMP-SMX was withdrawn in nine treatment episodes by the treating physician. One serious ADR occurred in the non-high-risk subgroup with a hospitalization for pancytopenia, which rapidly improved after discontinuation of TMP-SMX. No significant difference was observed in the incidence of ADR according to the underlying rheumatic disease (data not shown).

**Table 4:** Incidence of adverse drug reactions caused by prophylactic trimethoprim–sulfamethoxazole

	Number of cases^a^	Incidence rate (95% CI)^b^
Mild-to-moderate adverse drug reactions	11	30.7 (15.3–55.0)
Anemia	1	2.8 (0.1–15.6)
Thrombocytopenia	2	5.6 (0.7–20.2)
LFT abnormality	3	8.4 (1.7–24.5)
Skin rash	2	5.6 (0.7–20.2)
Azotemia	1	2.8 (0.1–15.6)
Others^c^	2	5.6 (0.7–20.2)
Serious adverse drug reactions^d^	1	2.8 (0.1–15.6)
Pancytopenia^d^	1	2.8 (0.1–15.6)
Stevens–Johnson syndrome	0	NA

*CI* confidence interval, *LFT* liver function test, *NA* non-applicable

^a^Total observation period was 31.0 person-years for 45 episodes

^b^Rate per 100 person-years

^c^Including headache (*n* = 1) and tingling sensation (*n* = 1)

^d^Occurred in the non-high-risk subgroup

On the basis of the one case of serious ADR in the prophylaxis group, the number needed to harm (NNH) was 45 (95% CI, 15 to ∞ [95% CI of absolute risk increasing was − 2.1 to 6.5]). In the whole population in the medium-steroid-dose group, the number needed to treat (NNT) with TMP-SMX to prevent one case of PCP was 204 (95% CI, 109–1624). However, in the high-risk subgroup, the NNT was 31 (95% CI, 17–226).

## Discussion

Glucocorticoid treatment is an essential part of the therapy for many rheumatic diseases, but is also an important risk factor for PCP. The association of high mortality with PCP means that a universal recommendation regarding PCP prophylaxis is important for the improvement of treatment outcomes, and this goal requires the establishment of an evidence-based threshold for the commencement of prophylaxis. To the best of our knowledge, this is the first study to investigate the incidence of PCP and its risk factors in patients with rheumatic diseases who received prolonged, medium-dose steroid treatment (≥ 15 to < 30 mg/day prednisone or equivalent).

In this study, the 1-year IR of PCP in the medium-dose group was 0.5 per 100 person-years, which was prominently lower than that in the high-dose group. However, the IR of PCP was highly variable and was affected by whether a patient had lymphopenia and/or had received concomitant steroid-pulse treatment at baseline. No PCP cases were associated with treatment episodes without these risk factors, suggesting that prolonged, medium-dose steroid treatment alone does not lead to sufficient immunosuppression for the occurrence of PCP. Previous experimental data have also demonstrated that medium-dose steroid treatment (unlike high-dose treatment) does not fully saturate the glucocorticoid receptor which leads to immunosuppression by genomic effect [16, 21, 22]. In addition, the most previously reported cases of PCP in patients without high doses of steroids were associated with other immunosuppressive conditions, such as lymphopenia, or with the concomitant use of other immunosuppressants [12, 23–25]. Taken together, our results suggest that a prolonged (≥ 4 weeks), 30-mg/day dosage of prednisone (or equivalent) is a relevant threshold for justification of PCP prophylaxis in patients with rheumatic disease who do not have any other risk factors.

Notably, our results demonstrated that treatment episodes for patients receiving prolonged medium-dose glucocorticoids with at least one risk factor (in particular, concomitant steroid pulse) were associated with a comparable PCP incidence to that in the high-dose group. This result was contrary to that of our previous study, in which steroid-pulse treatment did not increase the risk of PCP in patients receiving prolonged high-dose steroids [11]. Results from previous studies of PCP in patients with rheumatic diseases showed that it usually occurs after > 4 weeks of steroid treatment [6, 25, 26], suggesting that both duration and dose of the steroid treatment are important predisposing factors for PCP occurrence. Therefore, pulse treatment could increase the risk of PCP by a different mechanism from that of prolonged high-dose steroid treatment. This mechanism might involve acute immunosuppression and subsequent rapid immune reconstitution after the pulse treatment [27]. It is also supported by some previous reports showing that rapid reduction of immunosuppressive agents is significantly associated with PCP in patients without HIV [9, 28, 29].

In the current study, PCP incidence in the high-risk subgroup was comparable to that in the high-dose group, but TMP-SMX prophylaxis did not significantly reduce the 1-year PCP incidence. The number of treatment episodes with prophylaxis in the high-risk subgroup was small (*n* = 18), so this study did not have adequate power to demonstrate efficacy of prophylaxis. However, in a risk–benefit assessment, the NNT to prevent one case of PCP in the high-risk group was numerically lower than the NNH for serious ADR. Although the NNH was calculated for the entire medium-dose group (because the only serious ADR occurred in the non-high-risk subgroup), this result suggests that the clinical benefit of prophylactic TMP-SMX could outweigh the risk for serious ADR in high-risk patients. However, considering the wide ranges of 95% CI of NNT and NNH, this result should be replicated in the future studies.

It is also interesting that most ADRs related to TMP-SMX had mild-to-moderate severity, but prophylaxis was withdrawn in 75% of the episodes in which these ADRs occurred. It could be attributed to the physicians’ concern for possible progression to serious ADR such as Stevens–Johns syndrome. Results from previous studies also have reported a high incidence of ADR related to TMP-SMX, especially in patients with SLE [30, 31]. However, considering the high mortality associated with PCP in patients with rheumatic diseases, risk–benefit analysis of prophylaxis should be based not only on the incidence of ADRs, but also on their severity.

Some limitations of this study should be considered. First, the number of treatment episodes with prophylaxis was too small to precisely estimate the prophylactic efficacy of TMP-SMX. It also led to wide 95% confidence interval of both NNT and NNH, which make the result less powered. Therefore, it should be confirmed in the future studies including greater number of patients with prophylaxis. However, our data clearly showed that PCP did not occur despite large-scale observation in the treatment episodes where patients did not have risk factors. Second, although we defined the high-risk subgroups based on the LASSO selection method, it is uncertain whether these criteria can be generalized in other populations. Although all PCP cases occurred in the high-risk subgroup, the predictive performance should be validated in other populations. Third, because this study was retrospective, patients in the prophylaxis groups were more likely to have clinical factors that increase the risk for PCP (steroid pulse, interstitial lung disease, and numerically high cumulative steroid use before the baseline), which could lead to a biased result. However, this ‘confounding by indication’ biased away the result from showing a protective effect so true prophylaxis effect might be even better than estimated. In addition, change in the immunosuppressant use during the observation could be also different between the two groups. Although we showed that cumulative dose of steroid treatment between the two groups was comparable, difference in other immunosuppressive agent uses was not considered in the analysis. Finally, unmeasured confounders such as patients’ compliance cannot be completely adjusted by statistical manipulation.

## Conclusion

In conclusion, our results showed the IR of PCP in patients with rheumatic disease treated with various steroid dosages, and proposed threshold for steroid treatment in the presence or absence of other risk factors for which primary PCP prophylaxis can be justified. Although this result should be confirmed in future studies, it would be an important basis for a universal guideline regarding PCP prophylaxis in patients with rheumatic diseases.

## Supplementary information

**Additional file 1: Figure S1.** Flow chart of analysis in the study. **Figure S2.** Algorithm for detection of PCP cases in patients fulfilling the criteria for analysis. **Figure S3.** Algorithm for selection of treatment episodes. **Table S1.** Baseline^*^ characteristics of treatment episodes with medium-dose versus high-dose steroid treatment. **Table S2.** Clinical features of the five PCP cases in the study (medium-dose group) population. **Figure S4.** Determination of the optimal model to predict the pneumocystis pneumonia using the LASSO selection method. **Table S3.** Prophylactic effect of TMP-SMX on 1-year PCP incidence in various subgroups. (DOCX 1526 kb)

## Data Availability

All of the data supporting the conclusions of this article are included within the article.

## Abbreviations

ADR: Adverse drug reaction
CI: Confidence interval
EGPA: Eosinophilic granulomatosis with polyangiitis
GPA: Granulomatosis with polyangiitis
HIV: Human immunodeficiency virus
HR: Hazard ratio
ICD: International Classification of Diseases
ILD: Interstitial lung disease
IR: Incidence rate
MMF: Mycophenolate mofetil
MPA: Microscopic polyangiitis
MTX: Methotrexate
NNH: Number needed to harm
NNT: Number needed to treat
PCP: Pneumocystis pneumonia
SD: Standard deviation
SLE: Systemic lupus erythematosus
TMP-SMX: Trimethoprim–sulfamethoxazole
TNFi: Tumor necrosis factor inhibitor
