# Supplementary material for: Pneumocystis pneumonia in patients with rheumatic diseases receiving prolonged, non-high-dose steroids—clinical implication of primary prophylaxis using trimethoprim–sulfamethoxazole
Source: Arthritis Res Ther. 2019 Sep 14;21:207. doi: 10.1186/s13075-019-1996-6 (PMC6744623; doi:10.1186/s13075-019-1996-6)
Supplement: Supplementary file 1 — Additional file 1: Figure S1. Flow chart of analysis in the study. Figure S2. Algorithm for detection of PCP cases in patients fulfilling the criteria for analysis. Figure S3. Algorithm for selection of treatment episodes. Table S1. Baseline* characteristics of treatment episodes with medium-dose versus high-dose steroid treatment. Table S2. Clinical features of the five PCP cases in the study (medium-dose group) population. Figure S4. Determination of the optimal model to predict the pneumocystis pneumonia using the LASSO selection method. Table S3. Prophylactic effect of TMP-SMX on 1-year PCP incidence in various subgroups. (DOCX 1526 kb) [file 13075_2019_1996_MOESM1_ESM.docx]

**Supplementary Text.** ICD-10 codes for detection of treatment episodes in patients with rheumatic diseases.

Systemic lupus erythematosus (SLE; M32)

Systemic sclerosis (M34)

Dermatomyositis (M33.0, M33.1 and M33.9)

Polymyositis (M33.2)

Granulomatosis with polyangiitis (GPA; M31.3)

Microscopic polyangiitis (MPA; M31.7)

Eosinophilic granulomatosis with polyangiitis (EGPA; M30.1)

Polyarteritis nodosa (M30.0 and M30.8)

Rheumatoid arthritis (M05)

Adult-onset Still’s disease (AOSD; M06.1)

Behcet’s disease (M35.2)

Cryoglobulinaemic vasculitis (D89.1)

Ankylosing spondylitis (M45)

Temporal arteritis (M31.5 and M31.6)

Polymyalgia rheumatica (M35.3)

Takayasu’s arteritis (M31.4)

Relapsing polychondritis (M94.1)

Sjogren’s syndrome (M35.0)

Hypersensitivity vasculitis (M31.0)

Mixed connective tissue disease (M35.1)

IgG4-related disease (D89.8)

**Figure S1.** Flow chart of analysis in the study.

**Step 1. Capture of treatment episodes in different dose groups**

**Step 2. Calculate the 1-year incidence rate of PCP in each dose group**

**Step 3. If the 1-year incidence rate of PCP in the dose group was >0.1 per 100 person-years, each treatment episodes in this dose group was classified into one of the two groups according to whether the patient received primary TMP-SMX prophylaxis**


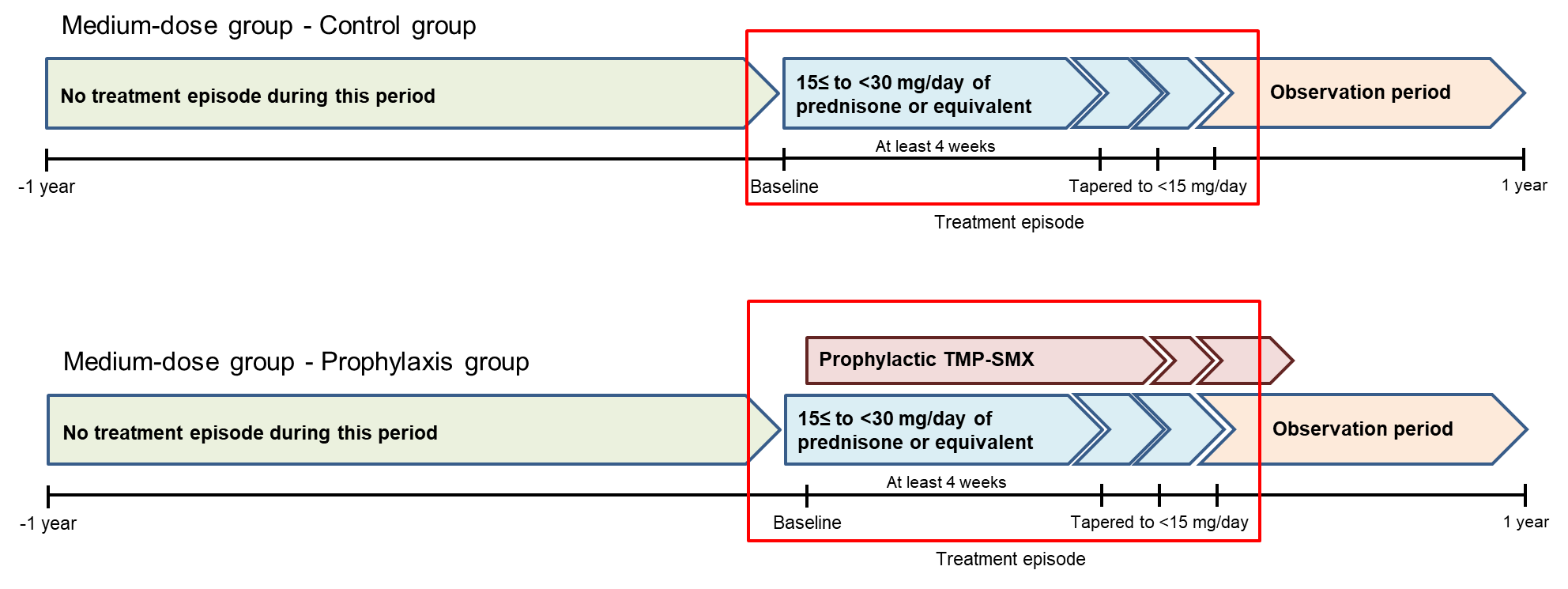

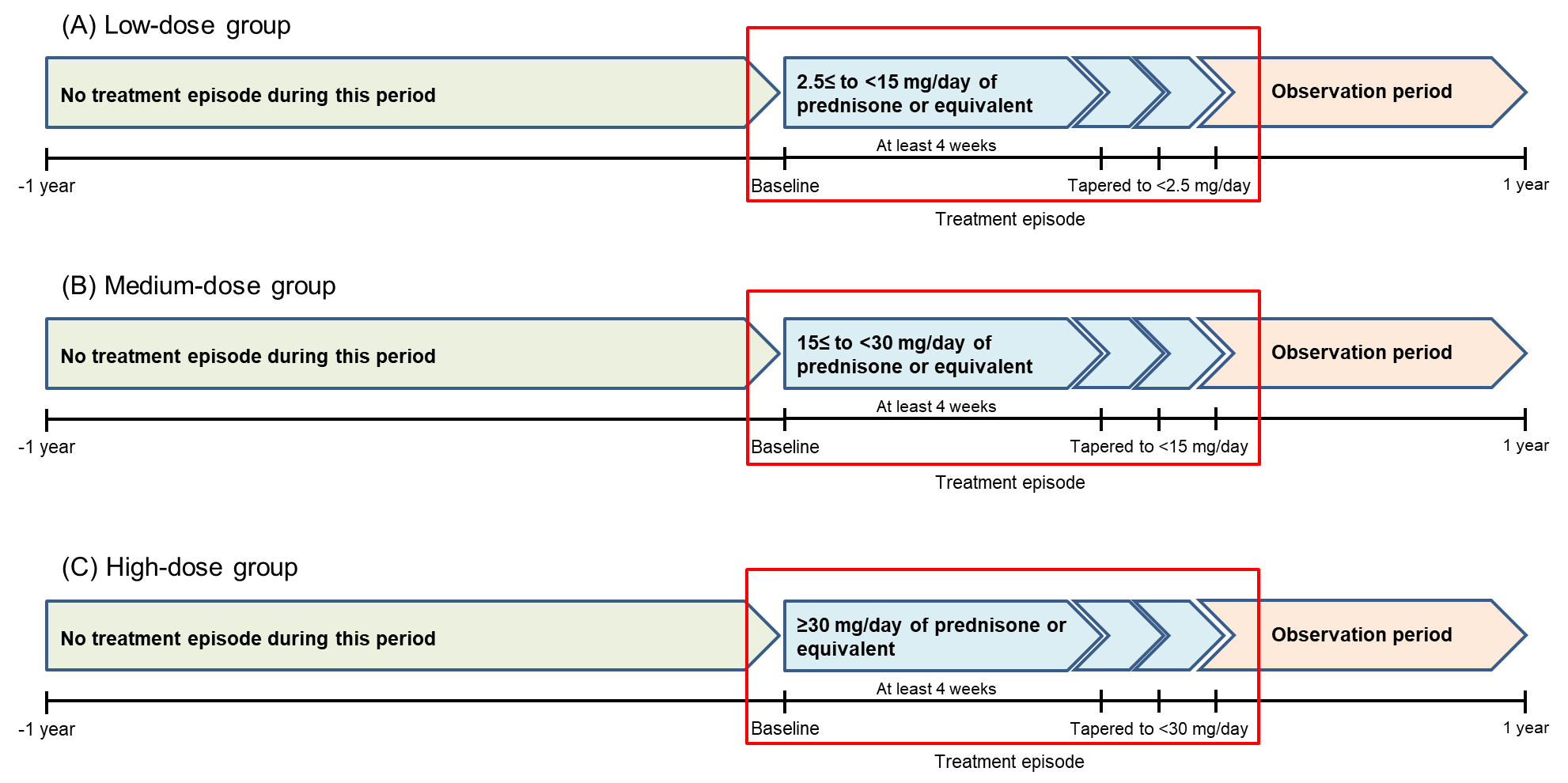


**Figure S2.** Algorithm for detection of PCP cases in patients fulfilling the criteria for analysis.


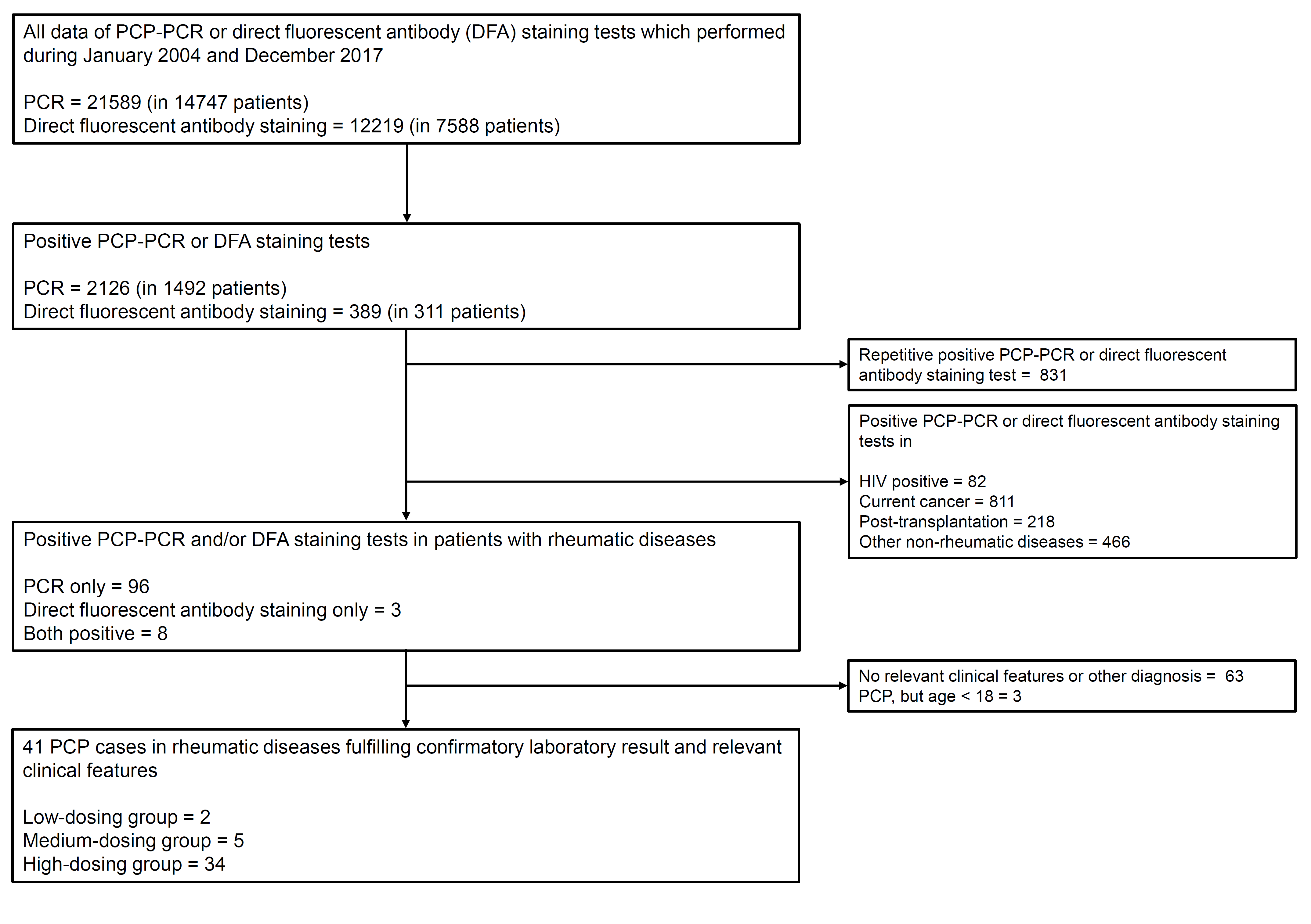


**Figure S3.** Algorithm for selection of treatment episodes.

**Table S1.** Baseline^*^ characteristics of treatment episodes with medium-dose versus high-dose steroid treatment

| (*n* = number of treatment episodes) | Medium-dose group  (*n* = 1065) | High-dose group  (*n* = 1666) | *p-*value |
| --- | --- | --- | --- |
| Male sex, *n* (%) | 296 (27.8) | 506 (30.4) | 0.149 |
| Age (years), mean (SD) | 43.4 (15.0) | 42.5 (15.7) | 0.129 |
| Disease duration (years), mean (SD) | 5.1 (4.5) | 3.1 (4.2) | <0.001 |
| Underlying disease |  |  |  |
| Systemic lupus erythematosus, *n* (%) | 473 (44.4) | 813 (48.8) | 0.025 |
| Systemic sclerosis, *n* (%)^¶^ | 22 (2.1) | 35 (2.1) | 0.950 |
| Dermatomyositis, *n* (%) | 59 (5.5) | 154 (9.2) | <0.001 |
| Polymyositis, *n* (%) | 57 (5.4) | 76 (4.6) | 0.349 |
| GPA, *n* (%) | 6 (0.6) | 59 (3.5) | <0.001 |
| MPA, *n* (%) | 3 (0.3) | 24 (1.4) | 0.003 |
| EGPA, *n* (%) | 29 (2.7) | 53 (3.2) | 0.494 |
| Polyarteritis nodosa, *n* (%) | 8 (0.8) | 30 (1.8) | 0.022 |
| Rheumatoid arthritis, *n* (%)^¶^ | 67 (6.3) | 79 (4.7) | 0.079 |
| Adult-onset Still’s disease, *n* (%) | 28 (2.6) | 42 (2.5) | 0.862 |
| Behcet’s disease, *n* (%) | 210 (19.7) | 211 (12.7) | <0.001 |
| Cryoglobulinaemic vasculitis, *n* (%) | 0 (0.0) | 3 (0.2) | 0.166 |
| Ankylosing spondylitis, *n* (%) | 16 (1.5) | 13 (0.8) | 0.073 |
| Primary Sjogren’s syndrome, *n* (%) | 14 (1.3) | 10 (0.6) | 0.051 |
| Relapsing polychondritis, *n* (%) | 12 (1.1) | 12 (0.7) | 0.267 |
| Giant-cell arteritis, *n* (%) | 2 (0.2) | 6 (0.4) | 0.416 |
| Polymyalgia rheumatica, *n* (%) | 21 (2.0) | 8 (0.5) | <0.001 |
| Takayasu’s arteritis, *n* (%) | 28 (2.6) | 29 (1.7) | 0.113 |
| Others, *n* (%)^†^ | 10 (0.9) | 9 (0.5) | 0.221 |
| Concomitant immunosuppressive treatment |  |  |  |
| Steroid-pulse treatment, *n* (%) | 54 (5.1) | 291 (17.5) | <0.001 |
| Oral cyclophosphamide, *n* (%) | 18 (1.7) | 84 (5.0) | <0.001 |
| Cyclophosphamide-pulse treatment, *n* (%) | 38 (3.6) | 169 (10.1) | <0.001 |
| Cumulative steroid dose, mean (SD)^‡^ | 680.7 (1306.2) | 1837.7 (1830.5) | <0.001 |
| Lymphopenia, *n* (%)^§^ | 131 (12.3) | 401 (24.1) | <0.001 |
| TMP-SMX prophylaxis | 45 (4.2) | 319 (19.1) | <0.001 |

GPA, granulomatosis with polyangiitis; MPA, microscopic polyangiitis; EGPA, eosinophilic granulomatosis with polyangiitis; PCP, pneumocystis pneumonia; SD, standard deviation; TMP-SMX, trimethoprim-sulfamethoxazole.

^*^The baseline date was defined as the day on which PCP prophylaxis (prophylaxis group) or medium/high-dose steroid (control group) was initiated.

^†^Including mixed connective tissue disorder, IgG4-related disease and hypersensitivity vasculitis.

^‡^Cumulative steroid (prednisone) dose during the previous 6 months.

^§^Defined as <800 lymphocytes per microliter.

^¶^The main reason for the use of high-dose steroids in these diseases was associated interstitial lung disease.

**Table S2.** Clinical features of the five PCP cases in the study (medium-dose group) population

| Case | Sex | Age | Underlying disease | Interval between baseline and PCP (days) | Lymphocyte count at baseline* | Lymphocyte count at PCP* | Steroid dose at PCP^†^ (mg) | Concomitant steroid pulse at baseline | Concomitant cyclophosphamide at baseline | PCP prophylaxis | PCP-related death |
| --- | --- | --- | --- | --- | --- | --- | --- | --- | --- | --- | --- |
| 1 | F | 54.5 | Systemic sclerosis | 79 | 261 | 505 | 15.0 | Yes | Yes | No | Yes |
| 2 | F | 78.2 | Rheumatoid arthritis | 45 | 349 | 166 | 15.0 | No | No | No | Yes |
| 3 | M | 32.2 | Dermatomyositis | 169 | 3120 | 746 | 15.0 | Yes | No | No | No |
| 4 | F | 28.4 | Systemic lupus erythematosus | 89 | 118 | 274 | 20.0 | Yes | No | No | No |
| 5 | F | 38.7 | Systemic lupus erythematosus | 38 | 1014 | 436 | 20.0 | Yes | Yes | No | No |

F, female; M, male; PCP, pneumocystis pneumonia.

*Indicates the lymphocyte counts per milliliter.

^†^Based on the dose of prednisone.

**Figure S4.** Determination of the optimal model to predict the pneumocystis pneumonia using the LASSO selection method. (A) Tuning parameter selection in the LASSO model. Vertical black lines bracket n values, where the model including steroid pulse and baseline lymphopenia provides its best fits to the data. (B) Coefficient trajectories produced by the regression analysis.

**
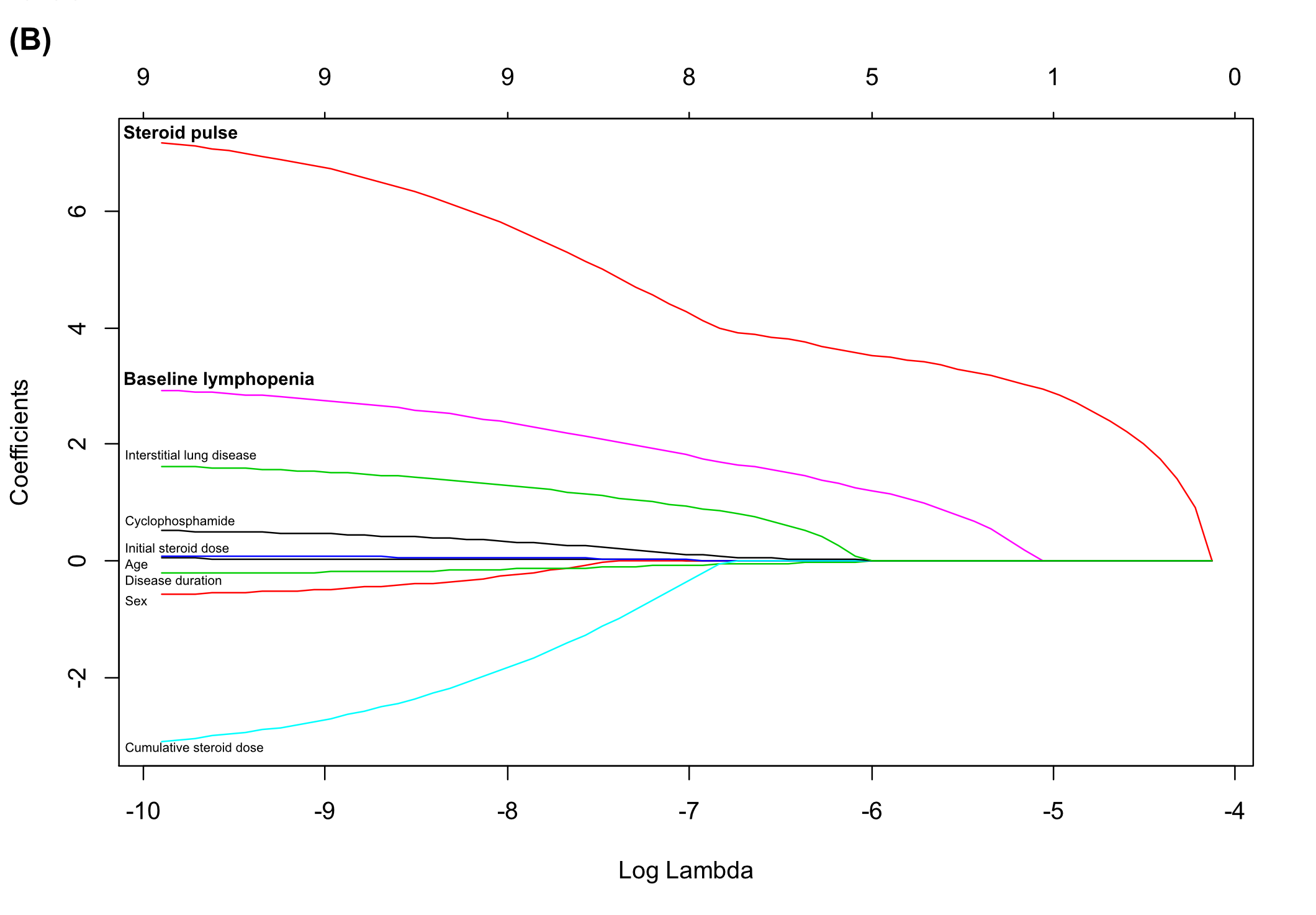
**

**Table S3.** Prophylactic effect of TMP-SMX on 1-year PCP incidence in various subgroups

| Subgroup | Adjusted HR^*^ | 95% profile-likelihood CI | *p-*value |
| --- | --- | --- | --- |
| Age ≥60 years (*n* = 147) | 3.19 | 3.30×10^-25^–1.28×10^7^ | 0.544 |
| SLE (*n* = 473) | 0.69 | 0.0005–4.01×10^20^ | 0.809 |
| Concomitant cyclophosphamide (*n* = 56) | 0.70 | 0.005–9.69 | 0.815 |
| Concomitant steroid pulse (*n* = 54) | 0.46 | 0.003–4.50 | 0.564 |
| High cumulative steroid^†^ (*n* = 249) | 0.58 | 0.004–6.53 | 0.707 |
| Baseline lymphopenia^‡^ (*n* = 131) | 1.11 | 0.008–13.15 | 0.947 |
| High-risk subgroup (*n* = 173) | 0.18 | 0.001–2.31 | 0.216 |

CI, confidence interval; HR, hazard ratio; PCP, pneumocystis pneumonia; SLE, systemic lupus erythematosus.

^*^HR was adjusted for clinical factors that showed significant association (*p* < 0.1) in the univariable analysis, and was adjusted for clustering.

^†^Defined as cumulative steroid dose >900 mg of prednisone or equivalent.

^‡^Defined as <800 lymphocytes per microliter.
